# DOPS (Direct Observation of Procedural Skills) in undergraduate skills-lab: Does it work? Analysis of skills-performance and curricular side effects

**DOI:** 10.3205/zma000987

**Published:** 2015-10-15

**Authors:** Christoph Profanter, Alexander Perathoner

**Affiliations:** 1 Medical University Innsbruck, Department of Visceral, Transplant and Thoracic Surgery, Innsbruck, Austria

**Keywords:** DOPS, skills lab, WBA (workplace based assessment), curricular side effects

## Abstract

**Objective: **Sufficient teaching and assessing clinical skills in the undergraduate setting becomes more and more important. In a surgical skills-lab course at the Medical University of Innsbruck fourth year students were teached with DOPS (direct observation of procedural skills). We analyzed whether DOPS worked or not in this setting, which performance levels could be reached compared to tutor teaching (one tutor, 5 students) and which curricular side effects could be observed.

**Methods: **In a prospective randomized trial in summer 2013 (April – June) four competence-level-based skills were teached in small groups during one week: surgical abdominal examination, urethral catheterization (phantom), rectal-digital examination (phantom), handling of central venous catheters. Group A was teached with DOPS, group B with a classical tutor system. Both groups underwent an OSCE (objective structured clinical examination) for assessment.

193 students were included in the study. Altogether 756 OSCE´s were carried out, 209 (27,6%) in the DOPS- and 547 (72,3%) in the tutor-group.

**Results:** Both groups reached high performance levels. In the first month there was a statistically significant difference (p<0,05) in performance of 95% positive OSCE items in the DOPS-group versus 88% in the tutor group. In the following months the performance rates showed no difference anymore and came to 90% in both groups.

In practical skills the analysis revealed a high correspondence between positive DOPS (92,4%) and OSCE (90,8%) results.

**Discussion:** As shown by our data DOPS furnish high performance of clinical skills and work well in the undergraduate setting. Due to the high correspondence of DOPS and OSCE results DOPS should be considered as preferred assessment tool in a students skills-lab.

The approximation of performance-rates within the months after initial superiority of DOPS could be explained by an interaction between DOPS and tutor system: DOPS elements seem to have improved tutoring and performance rates as well.

DOPS in students ‘skills-lab afford structured feedback and assessment without increased personnel and financial resources compared to classic small group training.

**Conclusion:** In summary, this study shows that DOPS represent an efficient method in teaching clinical skills. Their effects on didactic culture reach beyond the positive influence of performance rates.

## Zusammenfassung

**Zielsetzung: **Die suffiziente Vermittlung und Prüfung klinisch-praktischer Fertigkeiten bereits während des Medizinstudiums gewinnt zunehmend an Bedeutung. Im Rahmen des chirurgischen Pflichtpraktikums an der Medizinischen Universität Innsbruck wurde untersucht, ob das Teaching mittels DOPS im Skills-Lab Setting überhaupt funktioniert, zu welcher Performanz von klinischen Fertigkeiten DOPS (direct observation of procedural skills) im Vergleich zu einem Tutor-System (1 ärztlicher Tutor à 5 Studierende) führen und welche curricularen Side-Effects zu beobachten sind.

**Methoden:** Im Sommersemester 2013 (Monate April – Juni) wurden im Rahmen einer prospektiv randomisierten Studie 4 kompetenzlevelbasierte Skills mittels DOPS (Gruppe A) und einem klassischen Tutor System (Gruppe B) in einwöchigen Kleingruppen-Kursen gelehrt und mittels OSCE (objective structured clinical examination) geprüft:

Chirurgische Abdominaluntersuchung, Harnkatheteranlage (Phantom), rektal-digitale Untersuchung (Phantom), Handhabung zentralvenöser Katheter.

In die Studie wurden 193 Studierende inkludiert. Insgesamt wurden 756 Einzel-OSCE´s durchgeführt, davon entfielen auf die DOPS-Gruppe 209 (27,6%) und auf die Tutor-Gruppe 547 (72,3%).

**Ergebnisse: **Die Beobachtung der Performanz zeigt sehr gute Resultate in beiden Gruppen. Im ersten Monat wies die DOPS Gruppe gegenüber der Tutorgruppe einen statistisch signifikanten (p<0,05) Performanzunterschied von rund 95% versus 88% an vollständig erfüllten OSCE-Items auf. In den Folgemonaten glichen sich die Performanzen beider Gruppen weitgehend an und betrugen in beiden Gruppen rund 90%.

Bei den praktischen Fertigkeiten zeigte sich eine hohe Übereinstimmung zwischen DOPS- und OSCE-Resultaten (positive Ergebnisse: DOPS 92,4%, OSCE 90,8%).

**Diskussion: **Die Studiendaten zeigen, dass DOPS eine hohe Performanz klinischer Fertigkeiten erbringen und im studentischen Skills-Lab Setting gut funktionieren. Durch die hohe Übereinstimmung von DOPS- und OSCE-Ergebnissen im Assessment praktischer Fertigkeiten könnte man überlegen, DOPS auch als alleiniges Assessment-Tool im studentischen Skills-Lab einzusetzen.

Die zeitbedingte Annäherung der Performanzraten nach initialer Überlegenheit der DOPS-Gruppe könnte auf eine Wechselwirkung zwischen DOPS und klassischem Tutorsystem zurückzuführen sein: die DOPS-Elemente scheinen das Tutoring und die Performanz insgesamt verbessert zu haben.

Verglichen mit einem Kleingruppenunterricht bieten DOPS im studentischen Skills-Lab bei gleichem Personal- und Zeitaufwand zusätzlich strukturiertes Feedback und Assessment.

**Schlussfolgerung: **Zusammenfassend zeigt die vorliegende Studie, dass DOPS eine ressourcenschonende, effiziente Methode in der didaktischen Vermittlung klinisch-praktischer Fertigkeiten darstellt. Die Effekte der DOPS auf die universitären/klinischen Institutionen reichen weit über die unmittelbare positive Beeinflussung der Performanz hinaus.

## Introduction

Sufficient teaching and assessment of clinical skills already in the undergraduate setting becomes more and more important in view of changing requirements in health systems and planned reforms of medical curricula [1], [2], [3], [http://kpj.meduniwien.ac.at/fileadmin/kpj/oesterreichischer-kompetenzlevelkatalog-fuer-aerztliche-fertigkeiten.pdf]. For example in Austria a national competence level catalogue was recently implemented at all national medical universities [4]. Limitations of personnel and financial resources are challenging for academic teaching. Didactic methods will be evaluated in future not only in regard of performance but also of cost efficiency [5], [6], [7], [8].

Today there is much discussion in literature about efficiency of teaching and assessment methods [9], [10], [11], [12], [13]. MiniCEX (mini clinical evaluation exercise) and DOPS (direct observation of clinical skills) are used for some time as workplace based assessment (WBA) instruments in postgraduate settings [14], [15], [16], [17], [18], [19], [20], [21], [22], [23]. Recently, this didactic format is also used as WBA tool in final year undergraduate courses [24]. If DOPS is a reliable tool in teaching and assessment of clinical skills earlier in the curriculum, e.g. in the fourth year, is unknown [24]. Still open remains the question whether repeated assessment, e.g. with DOPS, is superior to conventional exercising in regard of performing clinical skills. Karpicke and Blunt stated in a prospective randomized study in the field of cognitive skills that retrieval practice produces more learning than elaborative studying [25]. Transferred to an undergraduate student´s skills lab DOPS would be the retrieval practice (repeated assessment) and the tutor teaching in small groups the elaborative learning. DOPS is well established as assessment tool in the context of workplace, especially in postgraduate settings [14], [15]. Simulation is a reliable instrument to create a workplace environment which is safe and provides a student centered setting [26].

In a prospective randomized study we analyzed the following questions:

- Does DOPS work in an undergraduate skills-labs setting?
- Does DOPS improve performance of clinical skills compared with tutor based teaching?
- How good is assessment quality of DOPS in this setting?
- Which curricular side effects result from the implementation of DOPS?

## Methods

### Setting/Study design

A prospective randomized trial at the Medical University of Innsbruck (MUI) from April to June 2013 was carried out in context of surgical practical studies in the eighth semester. The study was presented to the local ethics committee, which approved the study design and raised no objection to its realization. 

The multidisciplinary surgical practical studies are conducted primarily by the department of Visceral, Transplant and Thoracic Surgery (VTT), the following departments contribute to the studies in accordance to the department´s size: Department of Anesthesia and Intensive Care Medicine, Department of Trauma Surgery and Sports medicine, Department of Orthopedics and Orthopedic Surgery, Department of Heart Surgery, Department of Vascular Surgery, Department of Plastic, Reconstructive and Esthetic Surgery, Department of Urology, Department of Neurosurgery.

193 students out of a year cohort (n=258) were included in the study. The students were randomized into two groups. In both groups the same four competence-level-based skills were teached: surgical abdominal examination, urethral catheterization (phantom), rectal-digital examination (phantom), handling of central venous catheters. 

Group A was teached and assessed with DOPS only by lecturers of VTT. Group B was teached with a tutor system with lecturers of all other mentioned departments. Tutor teaching comprised small group lectures without a defined didactic method and one medical teacher present all the time. 

#### Randomization/Design of practical studies 

**Figure 1:**
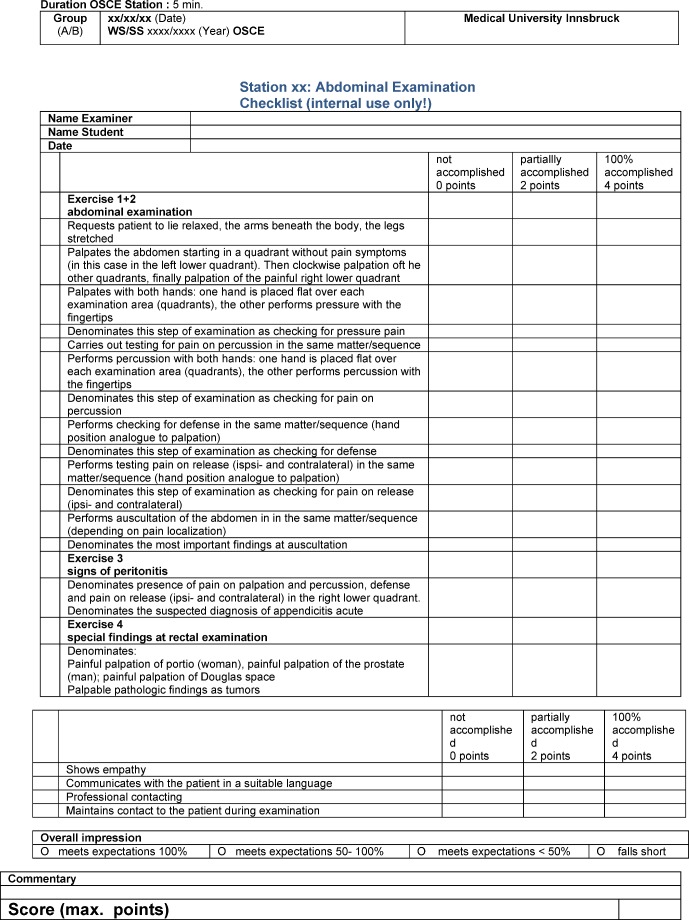
Example OSCE report form

The students were randomized in group A “DOPS” and Group B “Tutor” at the registration by chance. According to the chronological order of the registration odd numbers were randomized in the DOPS group, even numbers into the tutor group. The initial size of each group for analysis after randomization was n=109. The practical studies took place at the same time but in different rooms for each study group, which consisted of maximum five students. Each unit of the practical studies took five days, 90 minutes a day. At the first day all students got a theoretical lecture in all four skills based on detailed handouts for the lecturers. In the following days two, three and four the students were teached with DOPS or tutor system according to randomization. The students in the DOPS group were repeatedly assessed with the goal (minimum) 6 DOPS per student. Day five was dedicated to the OSCE assessment for all students. The OSCE comprised four five-minute stations in accordance to the teached skills. The OSCE test reports corresponded exactly to the mentioned handouts for the teachers (see Figure 1 (Fig. 1)).

#### Inclusion/Exclusion criteria

The registration for the practical studies (“Chirurgisches Praktikum”) was the only inclusion criterion. To warrant validity of data attention was carefully paid to avoid group shifts during the lecture days. Therefore, the following criteria for exclusion were defined in the study design:

1. Group shifting/students shifting form one group into the other (e.g. student of group A, four days in group A, one day in group B)
2. Students of DOPS group who missed 1 or more days (goal of 6 DOPS minimum not within reach)
3. Students in the Erasmus program, because they could not be obliged to undergo OSCE.

#### Skills/Materials/Assessment

The skills urethral catheterization and rectal-digital examination were teached and assessed on phantoms produced by Things and Limbs (Catheterization-Simulator, Rectal Examination Trainer MK2). The skill surgical abdominal examination was teached by the students as models themselves. For the corresponding OSCE station special trained student-actors were recruited. The skill handling of central venous catheters was teached and assessed on real devices that are used daily in the university hospital of Innsbruck. All OSCE examiners and actors were blinded regarding the randomization of the students.

The OSCE report forms were generated in the Innsbruck design (see Figure 1 (Fig. 1)). Content and layout of DOPS forms are in accordance with the original publications of Norcini and Darzi [14], [15]. 

#### Analysis/Statistics

Calculating OSCE results, the cut off for positive/negative rating was set between the category “completely accomplished” and “partially accomplished”. Thus, only item results from the category “completely accomplished” were rated as positive. The OSCE items were divided into two fractions according to the report form: skills and communication. The OSCE results were analyzed separately for each of the four skills and every month. The results were expressed in absolute numbers (points) and percent values.

Statistical analysis of OSCE results was performed by t-test. A p value <0,05 indicated a statistically significant difference.

DOPS items were also divided in two fractions – skills and communication. The skills fraction consisted of preparation/aftercare/certainty, technical performance, clinical judgment, organization and efficiency. The other items as professionalism were related to the communication fraction.

DOPS results in the categories satisfactory and superior were rated as positive and were summarized. The calculated percent values were compared with the OSCE results.

#### Instruction/Resources

All tutors and examiners were experienced residents or consultants/assistant professors and underwent specific OSCE teaching. Lecturers (and examiners) of VTT had an additional DOPS training. Special attention was directed to achieve a sufficient selectivity in assessment with OSCE and DOPS. The study design defined personnel and time related resources identically in both groups. Thus, the goal of 6 DOPS in summary or 2 DOPS each day was a result of this definition. Five students and 90 minutes per day amount to nine minutes for each DOPS as a total of observation and feedback time.

## Results

From the total number of students (n=193), n=52 (26,9%) were in the DOPS and 141 (73,1%) in the tutor group. 756 singular OSCE´s were performed, n=209 (27,6%) in the DOPS, n=547 (72,3%) in the tutor group. 180 (93,3%) students underwent all OSCE stations, 13 (6,7%) less than four. As these students could unmistakably be related to one of the groups (DOPS or tutor) in accordance to the criteria mentioned above, these OSCE results could be rated as well. 90,8% of the students in the DOPS group and 89,8% in the tutor group underwent all OSCE stations.

The size difference between the two groups (DOPS n=52, tutor n=141) can be explained by data adjustment in consequence of exclusion criteria: The most frequent reasons were groups shifting from DOPS to tutor and not suitable DOPS reports. Furthermore, some student groups randomized to DOPS did not participate in the practical studies at all and had to be cancelled.

### OSCE Skills

**Figure 2:**
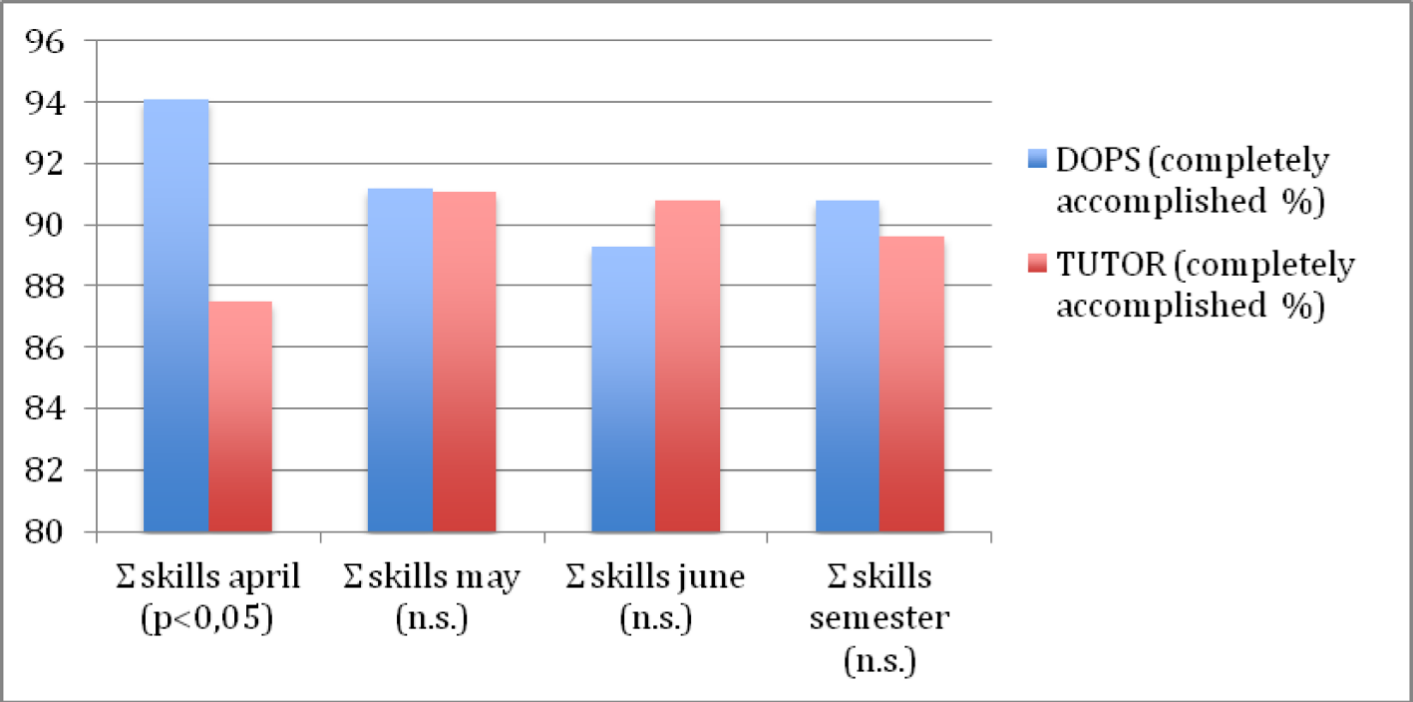
Results skills total

Overall, there cannot be found any difference in positive OSCE results between the study groups: 90,8% DOPS versus 89% tutor. Analyzing the monthly OSCE results leads to a different finding: in April the DOPS group was statistically significantly superior to tutor group with 94,1% versus 87,5% (p< 0,05). During the following months the performance rates approximated to each other. The rate of completely accomplished items was in May 91,2% (DOPS) and 91,1% (tutor), in June 89,3% versus 90,8% respectively (see Figure 2 (Fig. 2))

Also the analysis of the singular skills in the months May and June furnishes results close to each other. The percent mean values for DOPS and tutor were 91% versus 90,8% in May and 90% versus 90,5% in June. 

#### OSCE Communication

Generally, the performance in communication items was worse with 76,6% DOPS and 78,4% tutor (percent mean values over all months).

#### DOPS/Correspondence DOPS-OSCE

320 DOPS were performed at all. Analysis of skills related DOPS items amounted to 92,4% positive results. This value corresponds well with 90,8% positive OSCE results in the DOPS group.

We found a rate of 91,3% positive DOPS communication items. The correspondence with 76,6% positive OSCE results is substantial lower (see Table 1 (Tab. 1))

#### Resources

Due to the study design there was no difference regarding time and personnel resources. In both groups 5 students were assessed (DOPS) or supervised (tutor) by one teacher 90 minutes per day. The expenditure of time in personnel teaching was 6 hours. An instructor trained lecturers and examiners in groups with a maximum size of 15 people. Staff members of VTT and staff members of the other departments were always instructed separately.

## Discussion

DOPS as a teaching tool in an undergraduate setting achieve high performance levels of clinical skills. In our study, the performance rates in the DOPS group were found to be significantly (p<0,05) superior to the tutor system only in the first month of the semester (April). In the following months, May and June, and over the whole semester results showed no difference anyway (see Figure 2 (Fig. 2) and Table 1 (Tab. 1)) Thus, we conclude that DOPS works well in a student´s skills lab setting and scores at least equally with a tutoring in small groups and academic teachers.

Our data show a high correspondence between DOPS and OSCE results regarding skills. We venture to conclude that DOPS as assessment tool in students skills lab may be sufficient at least as pass and fail rating. Therefore, the OSCE could be dropped without diminishing the assessment quality. This would allow saving staff and time resources. According to the slogan “assessment time also is teaching time” either some more skills could be teached during the same time or the practical studies itself could be shortened e.g. four instead of five days. In our opinion this is an interesting curricular side effect that comes along with DOPS implementation in teaching undergraduates. Compared with a tutor system DOPS provide not only assessment but also structured feedback without generating additional costs. 

The approximation of skills performance rates after initial superiority of the DOPS group may be explained by interaction between DOPS and tutor system. Essential DOPS elements as structured feedback and repeated assessment were taken over from the teachers in the tutor system in the course of increasing mental embodiment. The study design provided, that only staff members of VTT supervise the DOPS groups and staff members of the associated departments the tutor groups. Only during the first weeks of these practical studies this mode could be realized. Later on, frequently staff members of VTT had to supervise also tutor groups out of organizational reasons. Nevertheless, both groups were always teached in different rooms.

In contrast to the positive results regarding skills as psychomotor competences, OSCE outcome concerning communicative items was substantial poorer. In both groups we found only about 77% positive results. This can be interpreted on the one hand as an achievement of adequate selectivity in assessment, which was an important topic in teachers’ instructions. On the other hand these data show weaknesses of students in this regard. Thus, this means a commission to focus more on these communicative aspects in the future within the skills lab setting. However, it remains open, which method is the best to achieve this improvement. Strictly speaking, we would have expected better results in the DOPS group. Probably, the intended focus on the psychomotor aspects in our setting and course design may be an explanation for the poorer OSCE results, but not for the also poor correspondence between DOPS and OSCE regarding these communicative aspects.

## Conclusion

In summary, our study shows that DOPS represents an efficient method in teaching clinical skills. Due to the high correspondence between DOPS and OSCE results regarding clinical skills it could be considered to carry out assessment in a student´s skills lab only with DOPS. Furthermore, DOPS implementation seems to have positive influence on the didactic culture of academic institutions.

## Competing interests

The authors declare that they have no competing interests.

## Figures and Tables

**Table 1:**
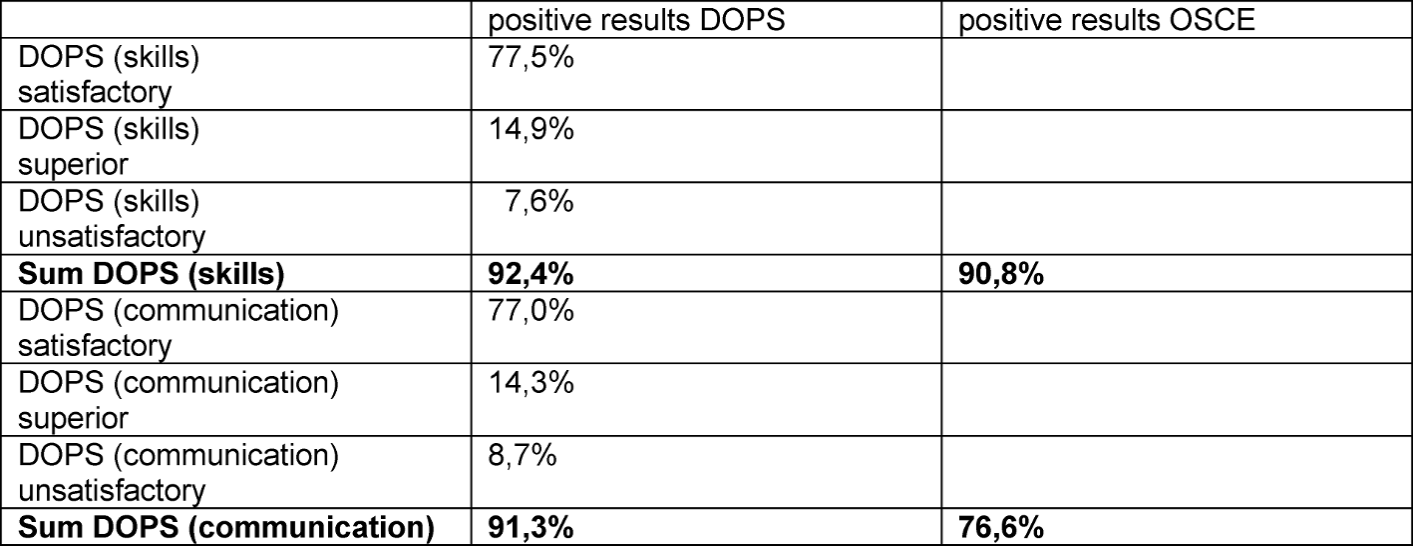
Correspondence positive DOPS and OSCE results
